# Superficial papilloma of the ovary

**DOI:** 10.1002/ccr3.5630

**Published:** 2022-03-24

**Authors:** Dimitrios Rafail Kalaitzopoulos, Markus Eberhard, Nicolas Samartzis

**Affiliations:** ^1^ Department of Gynecology and Obstetrics Cantonal Hospital Schaffhausen Schaffhausen Switzerland

**Keywords:** finding, laparoscopy, ovary, papilloma

## Abstract

Superficial papilloma of the ovary is a rare benign ovarian finding, which was first described back in 1895. Here, we present the intraoperative and histological findings of a superficial papilloma of the ovary in a 59‐year‐old patient.

## Graphical abstract

Superficial papilloma of the ovary is a rare benign ovarian finding with similar appearance to borderline and malignant papillary ovarian tumors. We suggest a histological confirmation of unclear ovarian findings in order to avoid overtreatment, especially in women with desire of fertility preservation.

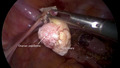

A 59‐year‐old patient was referred to our clinic for a laparoscopic hysterectomy for persistent hypermenorrhea. Preoperatively, physical and sonographic examination did not show any abnormality of the ovaries. During the operation, a papillary lesion of about 2 cm appeared on the surface of the left ovary, which we then completely excised (Figures 1 and 2).

**FIGURE 1:**
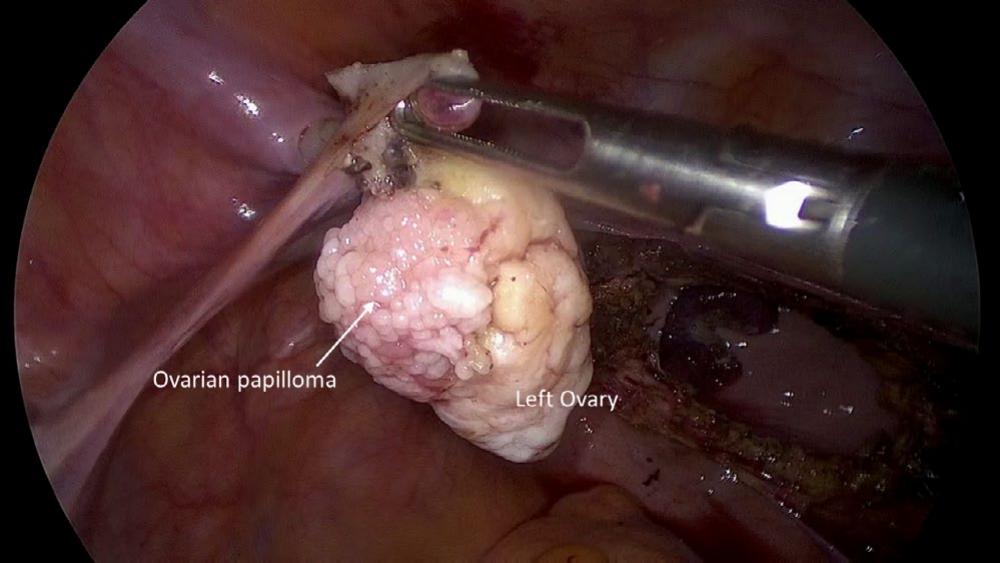
Intraoperative view of ovarian papilloma

**FIGURE 2:**
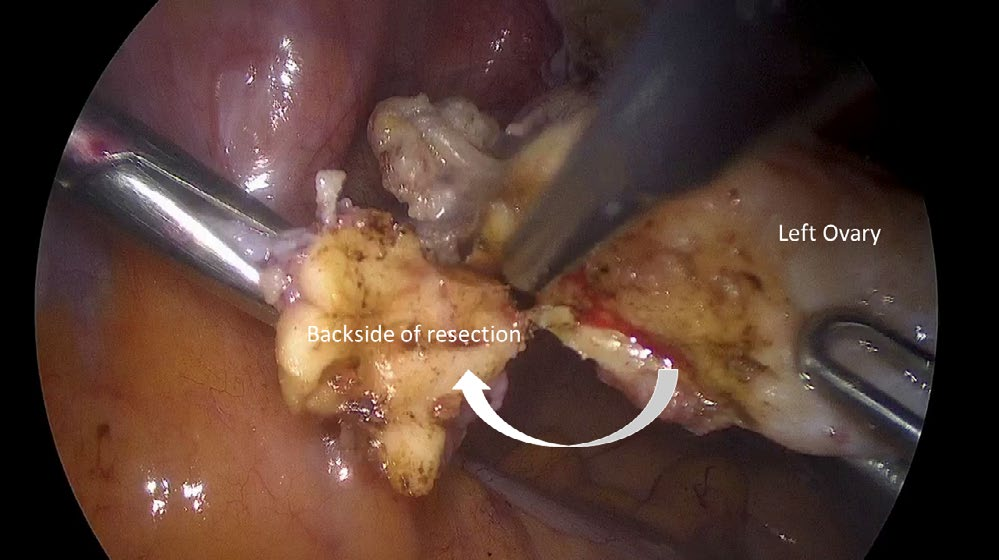
Intraoperative view during monopolar resection of the specimen

Histopathological examination showed an exophytic papillary tumor with serous epithelial cells and fibrotic stroma without significant mitosis or atypia, which was classified according to WHO classification as superficial papilloma of the ovary (Figure 3).

**FIGURE 3:**
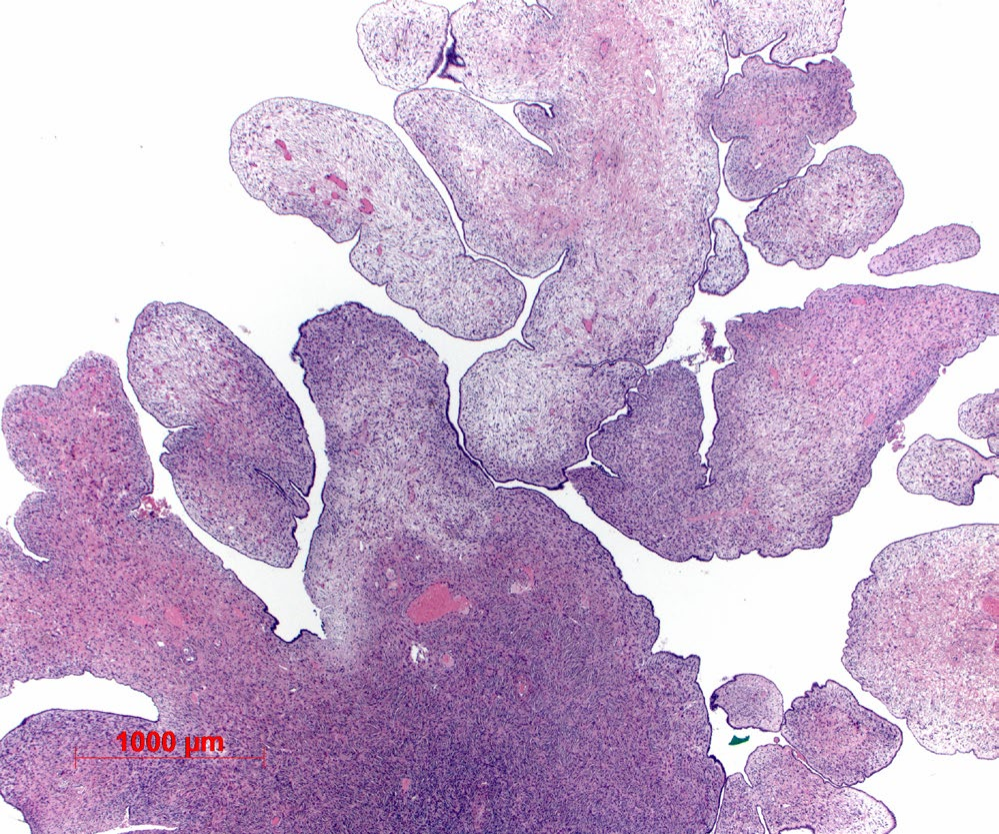
Histological image. Tumor consisting small papillary‐exophytic projections with fibrotic stroma (hematoxylin–eosin staining, magnification: 1:25)

Superficial papilloma of the ovary was first described by Freeborn back in 1895.^1^ As described in the literature, large lesions can lead to acute or chronic pelvic pain.

Kedzia et al. isolated HPV 16 DNA in a sample of ovarian papillomatosis and showed progesterone receptor‐positive tissue cells. Macroscopic similar exophytic proliferation are found in borderline and malignant ovarian papillary tumors.^2^

To sum up, superficial papilloma of the ovary is a rare benign ovarian lesion, which is usually an accidental intraoperative finding. Because of the similar appearance with borderline and malignant ovarian tumors, a histological confirmation is needed to avoid overtreatment, especially in women with desire of fertility preservation.

## CONFLICT OF INTEREST

The authors declare that they have no conflict of interest.

## AUTHOR CONTRIBUTIONS

DRK, NS, and ME conceived the presented idea. DRK wrote the manuscript. NS and ME supervised the final manuscript.

## ETHICAL APPROVAL

According to the Swiss law of research, no ethical approval for publishing of case reports is needed.

## CONSENT

Written informed consent has been obtained from the patient for publishing this report.

## Data Availability

The data that support the findings of this study are available on request from the corresponding author. The data are not publicly available due to privacy or ethical restrictions.
